# Cosmetic repair after sternochondroplasty by modified Ravitch procedure for pectus excavatum in a thin patient: A novel mesh sandwich technique

**DOI:** 10.1016/j.xjtc.2025.06.015

**Published:** 2025-06-26

**Authors:** Kim de Frémicourt, Jean-Marc Baste

**Affiliations:** a Department of Plastic and Reconstructive Surgery, ENT and Oncological Surgery, Henri Becquerel Anticancer Center, Rouen, France; b Department of Thoracic and Cardiac Surgery, Rouen University Hospital, Rouen, France

**Figure:**
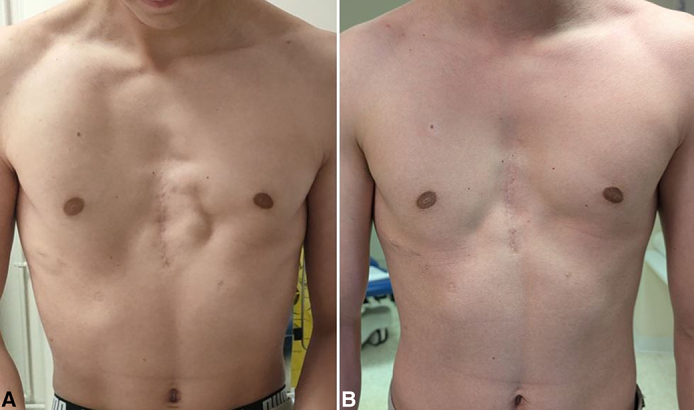
Cosmetic repair by a novel technique after sternochondroplasty for pectus excavatum.

**Central Message**

We describe a novel technique for correction of cosmetic and muscular pain sequalae after Ravitch procedure in a thin patient with retracted pectoralis major muscles.

The 2 most used techniques for pectus excavatum (PE) are the minimally invasive Nuss procedure and the open sternochondroplasty or modified-Ravitch procedure. PE correction should first be performed to improve cardiopulmonary function if altered.^1^ The cosmetic aspect comes secondarily; nevertheless, it is important for the patient's comfort and self-appreciation.

We describe an original technique for cosmetic and muscular pain sequalae improvement after sternochondroplasty for PE in a thin patient. Institutional review board/ethics review board approval was not required. No written informed consent was necessary for the publication of this case because all data have been sufficiently anonymized to protect the patient's identity and confidentiality.

## Surgical Technique

The patient was operated on during August 2022 for a sternochondroplasty for PE. The cartilage was removed from the perichondral sheaths at the level of the fourth to seventh rib by passing through the pectoralis major muscles (PMs) without detaching them from the sternum. After sternal osteotomy, a titanium bar was inserted retrosternally.

A few weeks after surgery, the patient presented with bilateral retracted PMs probably due to extensive muscle dissection and PM dehiscence. The lateral ectopic PM insertions were causing chronic pain and were visible at rest and during muscle contraction. Four months after surgery, the titanium bar was removed for skin effraction.

Because the patient still complained of chronic pain and mediocre cosmetic results even after bar withdrawal, he underwent reoperation 18 months after the primary surgery. Surgery was performed under general anesthesia. By the same vertical skin incision, the subcutaneous tissue was elevated. We noticed that the PMs were retracted and were inserted laterally on the periostea of the fourth to sixth rib, presumably because of muscle dehiscence caused by the initial surgery. The neocartilage formation was insufficient and presented abnormal mobility, probably due to the early bar withdrawal.

The muscle insertions on such structures most likely caused those contraction pain. Fixing back the PMs at this same state would not be efficient to ease pain because the mobility will reoccur. Additionally, the perichondria and neocartilages seemed not solid enough for solid muscle reinsertions. Also, we could not reapproximate the PMs in the midline because their retraction was too important.

**Figure 1:**
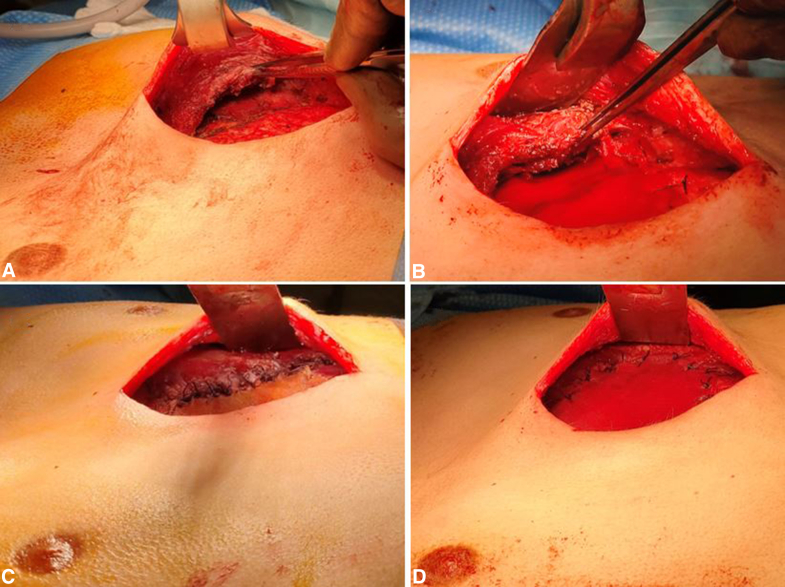
A, Elevation of the pectoralis major muscles (PMs). B, Submusclar mesh fixed on the thoracic cage. C, PM medial and inferior borders sutured on the submuscular mesh. D, Subcutaneous mesh fixed on top of the PMs.

**Figure 2:**
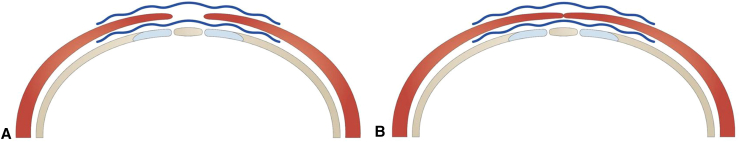
A, Preoperative. B, After 1-year follow-up.

Thus, we decided to elevate both PMs to place a polyglactin Vicryl (Ethicon) mesh submuscularly on the thoracic cage and fix it on the chest wall with several polyglactin 2.0 sutures (Figure 1, *A* and *B*). The PMs were then pulled as much as possible medially toward the sternum and attached thoroughly to the mesh (Figure 1, *C*). To reinforce the structure, reduce the risk of muscle dehiscence, and smooth the external aspect, another polyglactin mesh was laid subcutaneously, and fixed on top of the muscles (Figure 1, *D*). Because the PMs were between 2 layers of mesh, we named it the mesh sandwich technique (Figure 2, *A*).

**Figure 3:**
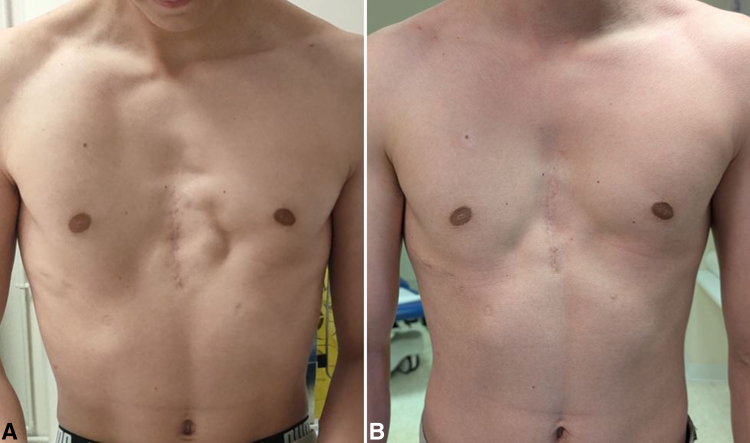
A, Mesh placement for revision surgery (*in blue: the meshes; in red: the pectoralis major muscles*). B, Mesh placement for primary sternochondroplasty with median muscle pexy.

A suction drain was placed, local anesthetic was infiltrated, and skin closure was performed. Because the subcutaneous undermining was quite extensive with thin tissues, a negative pressure wound therapy system (PICO; Smth + Nephew) was placed preventively for 7 days to optimize healing and reduce the risk of mesh exposure. The surgery lasted 2 hours and the patient was hospitalized for 2 days. The drain was removed 6 days later, and the patient was allowed to resume regular physical activity without limitations after 45 days. No skin dehiscence, seroma, nor infection occurred. At 1-year follow up, the patient did not have any more pain even during muscular contraction, no recurrence of muscle dehiscence was noticed, and the cosmetic aspect was highly improved (Figure 3).

## Discussion

Ravitch procedure is a reliable technique for PE treatment, especially for adults. Nevertheless, because the PMs are vastly dissected or partially detached from their sternal insertions, muscle dehiscence or retraction could occur, causing mediocre cosmetic results as well as muscle contraction pain.

For aesthetic correction, customized 3-dimensional implant and fat grafting are commonly used.^2^, ^3^, ^4^, ^5^ However, for this case, an implant was not a good indication because the chest wall irregularities were too superficial. Lipofilling could not be performed because the patient was too thin. Also, both techniques wouldn't have any effect on pain. This is why we sought to find a suitable covering to smooth the visual aspect as well as reinserting the PMs.

Herein, we used Vicryl mesh because it was the only mesh available on site. In future cases, a nonresorbable mesh could preferably be inserted submuscularly to improve the longevity of the muscle reattachments, although we did not notice any recurrence. Moreover, an acellular dermal matrix could be placed subcutaneously to enhance soft tissue trophicity. This approach could also be used at the same time as the Ravitch procedure to reduce the risk of revision for poor aesthetic outcome, which could be the case in thin patients (Figure 2, *B*).

## Conclusions

This original mesh sandwich technique could be an easy and efficient solution for PE cosmetic sequelae repair in thin patients.

## Conflict of Interest Statement

The authors reported no conflicts of interest.

The *Journal* policy requires editors and reviewers to disclose conflicts of interest and to decline handling or reviewing manuscripts for which they may have a conflict of interest. The editors and reviewers of this article have no conflicts of interest.
